# Long-term Immune Response to SARS-CoV-2 Infection Among Children and Adults After Mild Infection

**DOI:** 10.1001/jamanetworkopen.2022.21616

**Published:** 2022-07-13

**Authors:** Costanza Di Chiara, Anna Cantarutti, Paola Costenaro, Daniele Donà, Francesco Bonfante, Chiara Cosma, Martina Ferrarese, Sandra Cozzani, Maria Raffaella Petrara, Francesco Carmona, Cecilia Liberati, Paolo Palma, Giovanni Di Salvo, Anita De Rossi, Mario Plebani, Andrea Padoan, Carlo Giaquinto

**Affiliations:** 1 Division of Pediatric Infectious Diseases, Department for Women’s and Children’s Health, University of Padua, Padua, Italy; 2 Department of Statistics and Quantitative Methods, Division of Biostatistics, Epidemiology and Public Health, Laboratory of Healthcare Research and Pharmacoepidemiology, University of Milano-Bicocca, Milan, Italy; 3 Division of Comparative Biomedical Sciences, Istituto Zooprofilattico Sperimentale delle Venezie, Padua, Italy; 4 Department of Laboratory Medicine, University-Hospital of Padua, Padua, Italy; 5 Department of Surgery, Oncology and Gastroenterology, Section of Oncology and Immunology, University of Padova, Padua, Italy; 6 Istituto Oncologico Veneto - IRCCS, Padua, Italy; 7 Research Unit of Congenital and Perinatal Infections, Bambino Gesù Children's Hospital, Rome, Italy; 8 Department for Women’s and Children’s Health, University of Padua, Padua, Italy; 9 Department of Medicine, University of Padua, Padua, Italy

## Abstract

**Importance:**

Understanding the long-term immune response against SARS-CoV-2 infection in children is crucial to optimize vaccination strategies. Although it is known that SARS-CoV-2 antibodies may persist in adults 12 months after infection, data are limited in the pediatric population.

**Objective:**

To examine long-term anti–SARS-CoV-2 spike receptor-binding domain (S-RBD) IgG kinetics in children after SARS-CoV-2 infection.

**Design, Setting, and Participants:**

In this single-center, prospective cohort study, patients were enrolled consecutively from April 1, 2020, to August 31, 2021, at the COVID-19 Family Cluster Follow-up Clinic, Department of Women's and Children's Health, University Hospital of Padua. A cohort of 252 COVID-19 family clusters underwent serologic follow-up at 1 to 4, 5 to 10, and more than 10 months after infection with quantification of anti–S-RBD IgG by chemiluminescent immunoassay.

**Exposures:**

SARS-CoV-2 infection.

**Results:**

Among 902 study participants, 697 had confirmed SARS-CoV-2 infection, including 351 children or older siblings (mean [SD] age, 8.6 [5.1] years) and 346 parents (mean [SD] age, 42.5 [7.1] years). Among 697 cases, 674 (96.7%) were asymptomatic or mild. Children had significantly higher S-RBD IgG titers than older patients across all follow-up time points, with an overall median S-RBD IgG titer in patients younger than 3 years 5-fold higher than adults (304.8 [IQR, 139.0-516.6] kBAU/L vs 55.6 [24.2-136.0] kBAU/L, *P* < .001). Longitudinal analysis of 56 study participants sampled at least twice during follow-up demonstrated the persistence of antibodies up to 10 months from infection in all age classes, despite a progressive decline over time.

**Conclusions and Relevance:**

In this cohort study of Italian children and adults following SARS-CoV-2 infection different kinetics of SARS-CoV-2 antibodies were found across several age classes of individuals with asymptomatic or mild COVID-19, which could help in optimizing COVID-19 vaccination strategies and prevention policies. This work provides further evidence of sustained immune response in children up to 1 year after primary SARS-CoV-2 infection.

## Key Points

**Question:**

What are the long-term features of the immune response to SARS-CoV-2 in children compared with adults?

**Findings:**

In this cohort study of 252 family clusters with COVID-19, anti–SARS-CoV-2 spike receptor-binding domain IgG persisted until 12 months after infection in all age groups, showing significant higher antibody peaks for younger individuals at every follow-up time point. Children younger than 3 years were found to develop higher levels of binding antibodies compared with adults older than 18 years.

**Meaning:**

This study provided novel insights into the long-term features of the immune response to COVID-19 for different age classes, which could help in optimizing future COVID-19 vaccination strategies and prevention policies.

## Teaser

This cohort study of families with COVID-19 examines the long-term receptor-binding domain IgG kinetics in children compared with adults.

## Introduction

The ongoing SARS-CoV-2 pandemic has afflicted public health care systems worldwide. Vaccination is one of the most effective tools to achieve herd immunity in a short period. Consequently, a deeper understanding of the mechanisms related to long-term kinetics and durability of the immune response against SARS-CoV-2 is vital in optimizing vaccination strategies. In this respect, the anti–receptor-binding domain (RBD) antibodies against SARS-CoV-2’s spike (S) protein, with their strong positive correlation with the neutralizing antibodies (NAbs), represent a reproducible, cost-effective, and precise tool to define the quality of the host's immune response against the virus.^1,2,3,4,5,6^ Currently, scientific knowledge investigating the long-term persistence of anti–SARS-CoV-2 antibodies up to 12 months after the infection is mainly limited to adults,^7,8,9,10,11,12,13,14,15^ whereas a gap concerning the pediatric population, which plays an essential role in silently spreading the virus,^16,17^ still remains. To date, few studies^18,19,20^ have reported on the production of NAbs and anti–S-RBD IgG up to 8 to 12 months after infection in children who had recovered from mild or asymptomatic COVID-19. To gain a greater understanding of the immune response in children after SARS-CoV-2 infection, we examine the long-term anti–S-RBD IgG kinetics in a prospective cohort of COVID-19 family clusters mostly affected by asymptomatic or mild disease.

## Methods

### Study Design and Data Collection

We conducted a single-center, prospective cohort study of families, including children, older siblings, and their parents, who attended the COVID-19 Family Cluster Follow-up Clinic at the Department of Women's and Children's Health, University Hospital of Padua. From April 1, 2020, to August 31, 2021, families were enrolled 4 or more weeks after infection if they had children younger than 15 years and at least one family member with a history of COVID-19 infection. Exclusion criteria were receipt of the SARS-CoV-2 vaccine and classification as non–COVID-19 cases. Parents or legally authorized representatives were informed of the research proposal and provided their written informed consent. The study protocol was approved by the ethical committee of the University of Hospital of Padua.

At enrollment, a pediatrician (C.D.C., P.C., S.C., or C.L.) collected data on demographic characteristics, medical history, and vaccination status and performed a clinical evaluation. A blood sample was collected from all patients for characterization of the immunologic response to SARS-CoV-2. All patients with positive SARS-CoV-2 serologic test results at enrollment were followed up for longitudinal clinical and serologic evaluation with a minimum of 1 and a maximum of 3 collected blood samples at 1 to 4 and/or 5 to 9 and/or 10 or more months up to 18 months after baseline. Data on new contacts with confirmed or probable COVID-19 and confirmed SARS-CoV-2 subsequent infections were collected at each visit. Follow-up was interrupted if patients received any SARS-CoV-2 vaccine or in case of negative serologic test results. Data were anonymized and entered into a web-based database using the REDCap (Research Electronic Data Capture) platform (Vanderbilt University).

### Serologic Assays

Quantification of anti–SARS-CoV-2 S-RBD IgG antibodies was performed with commercially available chemiluminescent assays (Snibe Diagnostics, New Industries Biomedical Engineering Co Ltd [Snibe]). This method, previously validated elsewhere,^5^ quantitatively determined the IgG antibodies to the RBD portion of the SARS-CoV-2 spike protein. All analyses were conducted on MAGLUMI 2000 Plus (Snibe Diagnostics), with results expressed in kilo–binding antibody units per liter (kBAU/L), in accordance with the World Health Organization International Standard for anti–SARS-CoV-2 immunoglobulin. Samples recording titers greater than 4.33 kBAU/L were considered positive.

A high-throughput method for the plaque reduction neutralization test (PRNT) was used to quantify NAbs in serum samples for a subgroup of patients infected by SARS-CoV-2 within the first and second waves.^19,21^ The neutralization titer was defined as the reciprocal of the highest dilution resulting in a reduction of the control plaque count greater than 50% (PRNT_50_). Samples recording titers of 1:10 or greater were considered positive (eMethods in the Supplement).

### Case Identification and Definitions

Study participants were considered to have confirmed COVID-19 cases if they had a record of virologic positivity for SARS-CoV-2 by reverse transcriptase–polymerase chain reaction and/or tested positive on either of the 2 serologic tests adopted in this study. A confirmed SARS-CoV-2 subsequent infection was defined as the new detection of a positive SARS-CoV-2 virologic assay at nasopharyngeal swabbing, occurring 60 days or more after having recovered from a previous case of COVID-19 confirmed by negative virologic assay results.^22,23^ During the first wave of COVID-19 (from February 17 to September 18, 2020), all enrolled family members were systematically tested by both assays. However, during subsequent waves, a sudden increase in the enrollment rate brought us to reconsider the sustainability of applying both serologic assays, given the high economic and operational costs posed by the PRNT assay. Previous validation exercises had proven the high correlation between the 2 assays.^5^ For this reason, from March 26, 2021, all family members were tested for Snibe anti–SARS-CoV-2 S-RBD IgG levels.

Patients enrolled in the study were included in the statistical analysis if a defined baseline date was present. For symptomatic COVID-19 cases, the baseline date was defined as the first date between the onset of symptoms or the date of first positive SARS-CoV-2 molecular assay result. For asymptomatic cases, the baseline date was defined as the date of the first positive molecular assay result or, in those with only serologically confirmed COVID-19 and with negative or undetermined nasopharyngeal swab results, by the family outbreak temporal sequence, coinciding with the date of symptom onset in the family cluster. Infants younger than 6 months were included in the analysis only in case of virologic confirmation of SARS-CoV-2 infection at nasopharyngeal swabbing. COVID-19 severity was scored as mild, moderate, severe, critical, or multisystem inflammatory syndrome in children according to the World Health Organization classification.^24^ Patients who were asymptomatic and had no laboratory evidence of SARS-CoV-2 infection were considered non–COVID-19 cases. Three periods or COVID-19 waves were identified and defined as follows: a first wave from February 17 to September 18, 2020; a second wave from September 19, 2020, to February 18, 2021; and a third wave from February 19 to September 20, 2021.

### Statistical Analysis

Descriptive statistics, the χ^2^ test, the Fisher exact test, and a 2-tailed, unpaired *t* test were used for categorical or continuous covariates. The antibody titer response was assessed by comparing the median and the IQR of anti–SARS-CoV-2 S-RBD IgG values in the overall data set, including independent and participant-paired samples, and stratified by age classes (<3 years, 3-5 years, 6-11 years, 12-17 years, and ≥18 years) and by the time between serologic sampling and baseline, categorizing patients into 3 intervals, namely, 1 to 4, 5 to 9, and 10 or more months. The Kruskal-Wallis test was performed accordingly.

Longitudinal analysis of time between serologic sampling and baseline was conducted on participant-paired plasma from a subcohort of COVID-19 cases tested at least twice for S-RBD IgG titers, stratified by age classes (<6, 6-17, and ≥18 years). The Wilcoxon rank-sum test was performed accordingly. Linear regression analysis was used to assess the association between anti–SARS-CoV-2 S-RBD IgG and NAbs, using the log_2_ of both variables given data skew. Despite the transformation of the variables into logarithm, the strength of the associations between variables was assessed by the Spearman correlation coefficient and its relative *P* value.

The use of the robust variance estimator to account for correlations within patients with multiple blood samplings did not change the CIs considerably in the unadjusted analyses, so correlation structures were omitted from all analyses. Analyses were performed using the SAS software, version 9.4 (SAS Institute Inc). Statistical significance was set at *P* < .05. All *P* values were 2-sided. Graphs were made using GraphPad Prism, version 9.2 (GraphPad Software). This study followed the Strengthening the Reporting of Observational Studies in Epidemiology (STROBE) reporting guideline.

## Results

We prospectively evaluated 252 family clusters with COVID-19, for a total of 902 individuals. We excluded from the analyses 25 patients (2.8%) who had received at least 1 dose of SARS-CoV-2 vaccine before the first serologic follow-up and 180 patients (20.0%) defined as non–COVID-19 cases. A total of 575 patients (63.7%) who tested positive for SARS-CoV-2 by reverse transcriptase–polymerase chain reaction, together with 122 individuals (17.5%) who had no record of virologic positivity but showed evidence of seropositivity, were considered COVID-19 cases (eFigure 1 in the Supplement) and were included in the analysis. As a result, 697 patients with confirmed SARS-CoV-2 infection were analyzed, including 351 children or older siblings (mean [SD] age, 8.6 [5.1] years) and 346 parents (mean [SD] age, 42.5 [7.1] years). Among 697 cases, 674 (96.7%) were asymptomatic or mild. Descriptive characteristics of the patients and additional information on baseline time definition are reported in the eTable and eFigure 2 in the Supplement.

### Correlation Between Anti–SARS-CoV-2 S-RBD IgG and NAbs

**Figure 1.**
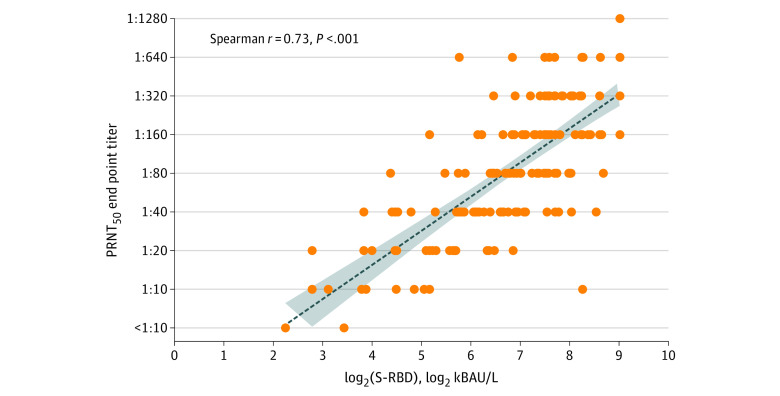
Correlation Between Spike Receptor-Binding Domain (S-RBD) IgG and Neutralizing Antibody Titers in 139 Patients Analyzed Simultaneously With Both Methods (172 Serum Samples) The dotted line represents the fitted line plot with 95% CIs. kBAU indicates kilo–binding antibody units; PRNT_50_, highest dilution resulting in a reduction of the control plaque count greater than 50% on the Plaque Reduction Neutralization Test.

From the 139 individuals who were tested in parallel with both serologic tests used in the study, a total of 172 samples were available for estimating the correlation between anti–SARS-CoV-2 S-RBD IgG and NAbs, detected by chemiluminescent immunoassay and PRNT_50_, respectively. Overall, in the linear regression model, a positive correlation was found between PRNT_50_ log titers and log_2_ S-RBD IgG titers (*R*^2^ = 0.47, ρ = 0.73, *P* < .001) (Figure 1). A similar correlation between PRNT_50_ log titers and log_2_ S-RBD IgG was observed when samples were stratified according to follow-up time points and age classes.

### Long-term Kinetics of Anti–SARS-CoV-2 S-RBD IgG

A total of 659 study participants had at least 1 anti–SARS-CoV-2 S-RBD IgG titer performed after infection. During follow-up, 657 (99.7%) still recorded positive titer results, whereas 2 of 659 patients (0.3%) with confirmed COVID-19 had negative antibody titer results after 64 and 556 days from baseline, respectively (Table 1; eFigures 3 and 4 in the Supplement).

**Table 1.** Serologic Data of 769 Serum Samples Obtained From 659 Individuals With Confirmed COVID-19 Among Different Age Classes, Overall and Stratified by Time From Baseline^a^

Variable	Anti-RBD, median (IQR), kBAU/L			
	All data	1-4 mo from onset	5-9 mo from onset	>10 mo from onset
Age class, y				
<3	304.83 (139.0-519.6)	342.8 (179.5-519.6)	284.3 (162.5-519.6)	146.2 (62.8-231.2)
≤3	169.3 (103.1-277.1)	234.6 (113.5-347.9)	118.2 (70.6-192.5)	115.6 (45.9-160.6)
≤6	126.2 (74.0-207.8)	164.1 (79.1-236)	119.7 (77.4-165.2)	90.6 (62.4-111.8)
≤12-18	98.2 (44.7-169.0)	103.1 (46.3-170.2)	89.6 (45.9-170.2)	48.6 (18.1-95.7)
≥18	55.6 (24.2-136.0)	64.5 (26.2-140.9)	49.8 (22.5-114.7)	36.7 (13.5-108.5)
*P* value^b^	<.001	<.001	<.001	.02

Abbreviations: kBAU/L, kilo–binding antibody units per liter; RBD, receptor-binding domain.

^a^ Serum samples at the last time point for 17 people whose last S-RBD IgG titer was higher than the previous one were excluded from the analysis.

^b^ Kruskal-Wallis test.

During follow-up visits, none of the patients reported exposure to other patients with COVID-19 or a subsequent confirmed SARS-CoV-2 infection. However, we recorded an unexpected increase in S-RBD IgG titer for 17 patients. Considering the possibility of an unknown exposure to SARS-CoV-2, the last serum samples from these 17 patients were excluded from the analysis.

**Figure 2.**
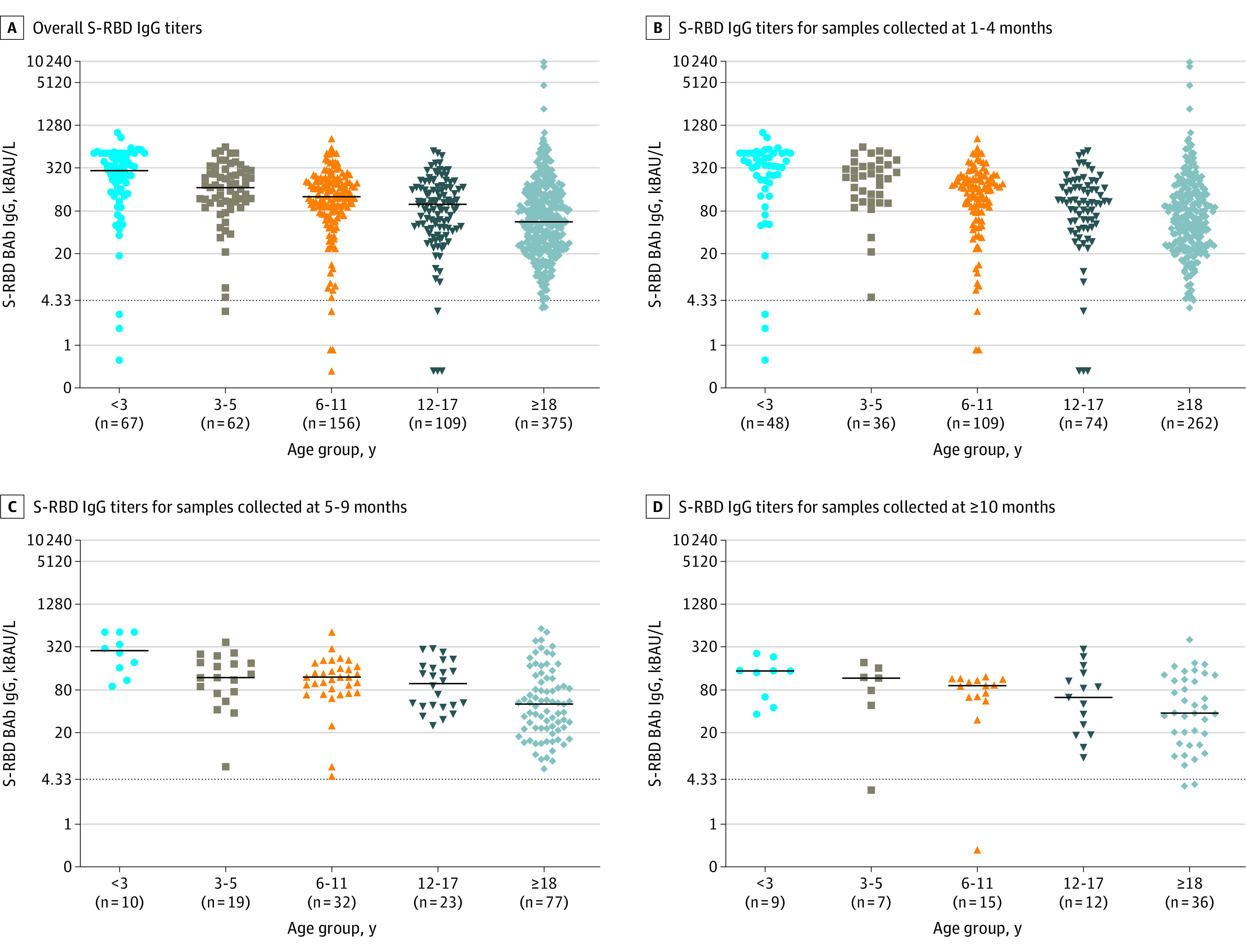
Distribution of Spike Receptor-Binding Domain (S-RBD) IgG Titers Note the progressive decrease of median antibody titers from children younger than 3 years to adults (age ≥18 years). Lines and whiskers represent medians and IQRs. The dotted line corresponds to the assay cutoff for discriminating positive from negative samples. BAb indicates early-binding antibody; kBAU, kilo–binding antibody units.

To better assess the association of age with the immunologic response, we analyzed 769 samples collected at 1 to 4 months (529 samples), 5 to 9 months (161 samples), and 10 or more months (79 samples) from baseline, stratifying among 5 classes of age (<3, 3-5, 6-11, 12-17, and ≥18 years of age) (Table 1 and Figure 2; eFigures 3 and 4 in the Supplement). The S-RBD IgG titers differed among age classes (Table 1 and Figure 2; eFigures 3and 4 in the Supplement). Overall, higher levels of antibodies were observed among younger children compared with older children, adolescents, and adults, with an overall median S-RBD IgG titer in patients younger than 3 years 5-fold higher than adults (304.8 [IQR, 139.0-516.6] kBAU/L vs 55.6 [24.2-136.0] kBAU/L, *P* < .001) (Table 1 and Figure 2; eFigures 3and 4 in the Supplement).

**Figure 3.**
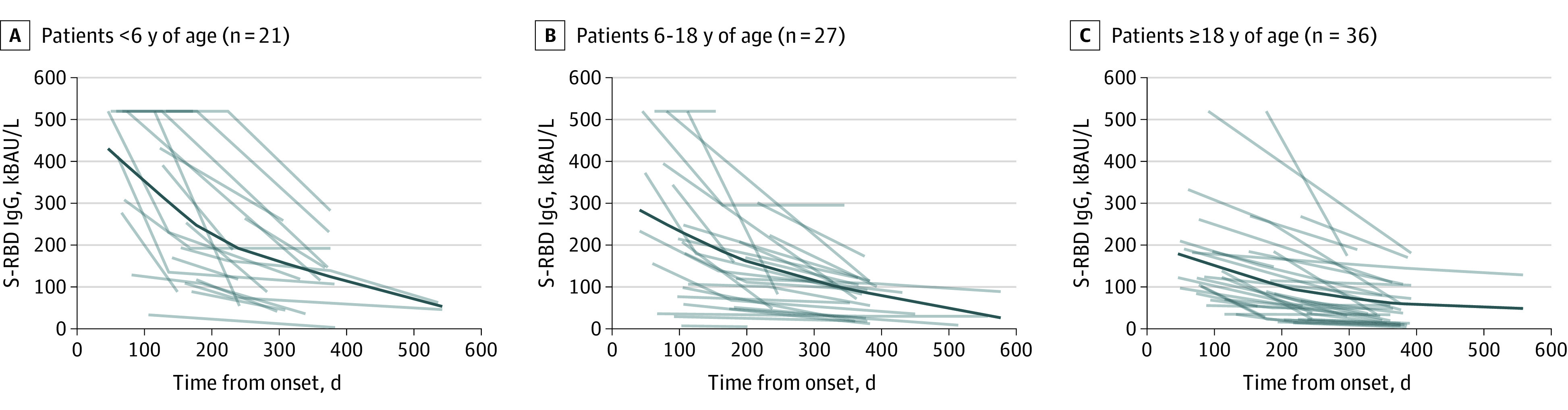
Individual Kinetics of Spike Receptor-Binding Domain (S-RBD) IgG Titers in Patients With At Least 2 Time Points of Follow-up Regardless of the Time of the First Serum Collection According to Age Groups and Collection Time (n = 194 Serum Samples) The dark blue lines represent the estimated antibody titer kinetics.

Differences in S-RBD IgG titers among all age classes, with younger children having significantly higher levels of antibodies, were also observed when samples were stratified by time of collection (at 1-4 months from infection, IgG anti-RBD levels ranged from 342.8 to 64.5 kBAU/L [*P* < .001]; at 5-9 months from infection, IgG anti-RBD levels ranged from 284.3 to 49.8 kBAU/L [*P* < .001]; and at ≥10 months from infection, IgG anti-RBD levels ranged from 146.2 to 36.7 kBAU/L [*P* = .02]) (Table 1 and Figure 3).

A longitudinal analysis was conducted on participant-paired plasma from a subcohort of 56 patients with COVID-19 tested at least twice for S-RBD IgG titers, with the first sample collected at 1 to 4 months from baseline. A first analysis was conducted on 31 patients who were sampled at a mean (SD) of 89.2 (38.6) and 199.2 (30.3) days from baseline, whereas a second analysis was conducted on 40 patients whose samples were collected at a mean (SD) of 81.9 (25.7) and 380 (47.7) days from baseline (referred to as medium and long intervals, respectively). Twenty-two patients were tested 3 times, contributing to both of these patient subgroups. Both analyses were stratified by 3 age subgroups: younger than 6 years, 6 to 18 years, and older than 18 years (Table 2).

**Table 2.** Participant-Paired Serologic Data of 56 Patients Who Were Sampled at Least Twice^a^

Age class, y	Time from baseline, mean (SD), d	Anti-RBD	
		Median (IQR) kBAU/L	*P* value^b^
First analysis			
<6			
First (1-4 mo)	98.0 (33.3)	455.1 (238.9-519.6)	.06
Intermediate (5-9 mo)	205.0 (31.7)	190.6 (113-519.6)	
6-18			
First (1-4 mo)	83.9 (28.6)	220.4 (155.9-519.6)	.004
Intermediate (5-9 mo)	195 (33.9)	106.1 (68.0-158.9)	
≥18			
First (1-4 mo)	87.2 (26.2)	104.8 (69.7-138.1)	<.001
Intermediate (5-9 mo)	198.8 (28.6)	52.0 (27.7-56.7)	
**Second analysis**			
<6			
First (1-4 mo)	80 (30.4)	475.6 (308.0-519.6)	.002
Late (≥10 mo)	360 (26)	132.7 (107-231.2)	
6-18			
First (1-4 mo)	82.9 (25)	180.3 (76.6-372.4)	<.001
Late (≥10 mo)	392.4 (53.3)	71.4 (29.9-113.7)	
≥18			
First (1-4 mo)	82.1 (24.8)	121.2 (68.4-209.6)	<.001
Late (≥10 mo)	380.9 (51.2)	48.1 (19.9-80.5)	

Abbreviations: kBAU/L, kilo–binding antibody units per liter; RBD, the receptor binding domain.

^a^ Overall, 31 patients were evaluated 1 to 4 months (mean [SD], 89.2 [38.6] days) and 5 to 9 months (mean [SD], 199.2 [30.3] days) from baseline, and 40 patients were evaluated 1 to 4 months (mean [SD], 81.9 [25.7] days) and 10 or more months (mean [SD], 380.0 [47.4] days) from baseline.

^b^ Wilcoxon signed-rank test.

All 3 age groups exhibited persistence of S-RBD IgG titers at both intervals. Nonetheless, a progressive decrease in antibody levels was observed among all age classes and ranged from 2.0- to 2.3-fold reductions for the medium intervals and 2.5- to 3.6-fold reductions for the long intervals (Table 2).

To better investigate the decay in antibodies across age groups, the same analysis was conducted on a subcohort of 84 patients with COVID-19 tested at least twice for S-RBD IgG titers, regardless of the time of the first serum collection, for a total of 194 samples. Tracing a theoretical line obtained considering differences among individual antibody titers of all patients, we observed that all 3 age groups exhibited progressive decay in antibody titers; the rate of antibody waning was more rapid during the first 200 days and progressively slower thereafter. Compared with adults and children 6 years or older, children younger than 6 years showed an apparently faster early waning of antibody titers (Figure 3). In addition, antibody titers remained detectable for 18 months (Figure 3).

## Discussion

In this cohort study, we evaluated the dynamic changes of the SARS-CoV-2 binding antibody titers in 252 family clusters mostly affected by asymptomatic or mild COVID-19 up to 12 months after initial infection. The findings suggest that anti–SARS-CoV-2 S-RBD IgG may persist more than a year from infection in all age groups, with antibody titers that inversely correlate with age.

This study strengthens and expands what we observed previously about the medium-term SARS-CoV-2 NAb response after COVID-19, analyzing preliminary data on the first 57 families affected by mild COVID-19 enrolled in our cohort.^19^ This study suggests that the magnitude of SARS-CoV-2 S-RBD IgG antibodies is higher among younger children compared with older siblings and adults at all follow-up points. Considering the 2 ends of the age spectrum of our cohort, we found that children younger than 3 years developed 5-fold higher levels of binding antibodies compared with individuals older than 18 years. These results align with prior studies^18,20,25,26^ using PRNT_50_ and surrogate neutralization–based assays describing higher antibody titer and neutralizing ability in children than adults.

Our results demonstrate different antibody titers among mildly affected age groups, suggesting that factors such as specific cellular responses, genetics, environment, and stochastic variables may contribute to the high variation in immune response between individuals, irrespective of disease severity.^27,28^ One study found that children had class-switched convergent cellular clones to SARS-CoV-2 before the pandemic, with weak cross-reactivity to other coronaviruses, whereas adult blood or tissues showed few clones.^29^ Another study^30^ reinforces our supposition, suggesting that infection in elderly patients is associated with antibodies targeting the cross-reactive S2 and NP proteins, whereas in children, the response is dominated by antibodies with high Fc-effector function targeting the immunodominant S1 protein of SARS-CoV-2. Conversely, Renk et al^20^ recently observed that the repeated exposure to previous endemic human coronaviruses did not impair the humoral response to SARS-CoV-2. Finally, given that our family clusters were likely exposed to similar environmental factors, genetic attributes may also contribute to the different potency and durability of humoral responses.^31^

Three studies contrast with our findings, reporting no differences in the expression of specific antibodies between age classes or lower neutralizing activity in children compared with adults.^32,33,34^ However, Márquez-González et al^32^ evaluated samples collected 3 weeks after infection, and 40% of pediatric patients were affected by malignant neoplasms at the time of COVID-19 diagnosis, implying a potential state of immunosuppression that may have altered the humoral response to infection. In the other studies,^33,34^ children were compared with mildly affected adults selected as plasma donors, meaning that potentially only hyperimmune adults were selected.

Our work suggests that anti–S-RBD antibodies persist up to 18 months after infection, even in children. Stratifying individuals by age groups, we demonstrated that both children and adults experienced a decrease in anti–S-RBD IgG levels mostly during the first 200 to 300 days from infection. Of interest, children younger than 6 years showed a faster waning of antibody titers and then reached a plateau without a conversion to negative results. These results are in line with previous studies^7,8,9,10,11,12,13,14,20,35,36,37,38,39^ conducted among both adults and children. In particular, Lau et al^39^ observed that antibodies were detectable by spike RBD enzyme-linked immunosorbent assays in 92.6% of serum samples at 200 to 386 days from infection, despite showing an assay-dependent kinetics of antibody levels.

The persistence of a detectable S-RBD IgG titer more than 10 months after infection was observed in all age groups, regardless of whether the titers decreased over time. Remarkably, children younger than 6 years exhibited a median (IQR) S-RBD IgG titer of 132.7 (107-231.2) kBAU/L at 373 (339-376) days from baseline, and only 2 patients had results that converted to negative.

A previous study^40^ estimated that the correlate of 50% protection from subsequent infection was 20% of the convalescent NAb titer. Relying on these findings, Lau et al^39^ estimated that the threshold for 50% protection from subsequent infection for PRNT_50_ was 1:25.9 (95% CI, 1:24.7-1:27.6). As previously estimated by Padoan et al,^5^ an S-RBD IgG titer greater than 70 kBAU/L is assumed to correspond to a PRNT_50_ titer greater than 1:20. In the current study, we demonstrated that the time-consuming PRNT_50_ correlated with the more available chemiluminescence assay on a large number of samples (n = 172), which could represent a promising open-access tool for estimation of serum’s neutralizing power. In line with these findings, our data indicate that children younger than 6 years might be protected from subsequent infection up to 1 year.

However, as different virus variants emerge, the level of protective immunity may be compromised. Although it was observed that antibodies showed strong cross-reactivity to different variants, including Beta, Delta, Gamma, and Mu, for more than 1 year after infection,^35^ future studies should also confirm the long-lasting response against Omicron. Moreover, to better understand the long-term persistence of immune protection against new emerging SARS-CoV-2 variants and to translate our data into estimations of immunity of children to subsequent infection, future research should include the evaluation of the longevity of B and T cells, which plays a key role in the human immune response. In fact, although we focused on the antibody responses to infection in this analysis, cellular immune responses are also likely to play an important role in protection against SARS-CoV-2 subsequent infection, as we and others have previously reported.^41^ Children had a higher absolute number of circulating T cells and a high proportion of naive T cells than adults, thus enabling an efficient adaptive immune response to previously unrecognized microbial antigens, which persisted until 6 months after infection.^42^

### Limitations

Our study has several limitations. First, operational challenges related to the pandemic restrictions affected organization and access to the clinic; therefore, patients were evaluated with different follow-up times, and for a proportion of patients, intermediate follow-up was missing. Second, the baseline of infection for those patients with COVID-19 without a positive nasopharyngeal swab result was identified through the only temporal reference to infection of the first symptomatic household and may be susceptible to temporal error. However, the initial temporal discrepancy, which may alter the evaluation of the acute phase of humoral response, was partially addressed by long-term follow-up.

## Conclusions

In this cohort study of Italian children and adults with SARS-CoV-2 infection, we found that anti–SARS-CoV-2 S-RBD IgG persisted until 12 months after infection in all age groups, with significant higher antibody peaks for younger individuals at every follow-up point. This study may provide an important basis to determine the schedule of COVID-19 vaccination in non–previously infected children and of booster immunization in pediatric patients who have already experienced COVID-19.

## Notes

Supplement. **eMethods.** Supplemental Methods **eFigure 1.** Family Clusters of COVID-19 Observed From April 1st, 2020 to August 31st, 2021, at the COVID-19 Follow-up Clinic of Our Institution **eFigure 2.** Criteria for the Definition of the Baseline Time for COVID-19 Cases **eFigure 3.** Distribution of S-RBD IgG Samples According to Time of Collection and Age Classes (n=769) **eFigure 4.** Distribution of S-RBD IgG Samples According to Age Classes **eTable.** Demographic and Clinical Characteristics of the Analyzed Population, Overall (n=876) and Stratified by Familiar Status as Children or Older Siblings (n=446) and Parents (n=431)
