# Supplementary material for: Long-term Immune Response to SARS-CoV-2 Infection Among Children and Adults After Mild Infection
Source: JAMA Netw Open. 2022 Jul 13;5(7):e2221616. doi: 10.1001/jamanetworkopen.2022.21616 (PMC9280400; doi:10.1001/jamanetworkopen.2022.21616)
Supplement: Supplement. — eMethods. Supplemental Methods eFigure 1. Family Clusters of COVID-19 Observed From April 1st, 2020 to August 31st, 2021, at the COVID-19 Follow-up Clinic of Our Institution eFigure 2. Criteria for the Definition of the Baseline Time for COVID-19 Cases eFigure 3. Distribution of S-RBD IgG Samples According to Time of Collection and Age Classes (n=769) eFigure 4. Distribution of S-RBD IgG Samples According to Age Classes eTable. Demographic and Clinical Characteristics of the Analyzed Population, Overall (n=876) and Stratified by Familiar Status as Children or Older Siblings (n=446) and Parents (n=431) [file jamanetwopen-e2221616-s001.pdf]

Supplementary Online Content

Di Chiara C, Cantarutti A, Costenaro P, et al. Long-term immune response to SARS-CoV-2 infection among children and adults after mild infection. *JAMA Netw Open*. 2022;5(7):e2221616. doi:10.1001/jamanetworkopen.2022.21616

eMethods. Supplemental Methods

eFigure 1. Family Clusters of COVID-19 Observed From April 1st, 2020 to August 31st, 2021, at the COVID-19 Follow-up Clinic of Our Institution

eFigure 2. Criteria for the Definition of the Baseline Time for COVID-19 Cases

eFigure 3. Distribution of S-RBD IgG Samples According to Time of Collection and Age Classes (n=769)

eFigure 4. Distribution of S-RBD IgG Samples According to Age Classes

eTable. Demographic and Clinical Characteristics of the Analyzed Population, Overall (n=876) and

Stratified by Familiar Status as Children or Older Siblings (n=446) and Parents (n=431)

This supplementary material has been provided by the authors to give readers additional information about their work.

© 2022 Di Chiara C et al. *JAMA Network Open.*

eMethods. Supplemental Methods

Serological assays

Blood samples were collected in EDTA-coated tubes to further separate cells and plasma by Ficoll procedure. Plasma and cellular samples were appropriately store at -80°C and liquid nitrogen, respectively, until use.

In a subgroup of 172 samples (from 139 patients), a high-throughput method for Plaque Reduction Neutralizing Test (PRNT) was used for the quantification of neutralizing antibodies in plasma samples[1,2]. Samples were heat-inactivated by incubation at 56°C for 30 min and 2-fold dilutions were prepared in Dulbecco modified Eagle medium (DMEM). The dilutions, mixed to a 1:1 ratio with a virus solution containing approximately 25 focus-forming units (FFUs) of SARS-CoV-2, were incubated for 1 h at 37 °C. Fifty microliters of the virus-serum mixtures were added to confluent monolayers of Vero E6 cells, in 96-wells plates and incubated for 1 h at 37 °C, in a 5% CO2 incubator. The inoculum was removed and 100 ml of overlay solution of Minimum essential medium (MEM), 2% fetal bovine serum (FBS), penicillin (100 U/ml), streptomycin (100 U/ml) and 0.8% carboxy methyl cellulose was added to each well. After a 26-h incubation, cells were fixed with a 4% paraformaldehyde (PFA) solution. Visualization of plaques was obtained with an immunocytochemical staining method using an anti-dsRNA monoclonal antibody (J2, 1:10,000; Sci- cons) for 1 hour, followed by 1 h incubation with peroxidase-labeled goat anti-mouse antibodies (1:1000; DAKO) and a 7 min incubation with the True Blue® (KPL) peroxidase substrate. FFUs were counted after acquisition of pictures on a flatbed scanner. Biosafety Level 3 laboratory setting was used for PRNT tests. The neutralization titer was defined as the reciprocal of the highest dilution resulting in a reduction of the control plaque count >50% (PRNT50). Samples recording titers equal to or above 1:10 were considered as positive according to a previous validation conducted on a panel of archived samples collected in 2018 in Italy[1].

eReferences

1. Padoan A, Bonfante F, Pagliari M, et al. Analytical and clinical performances of five immunoassays for the detection of SARS-CoV-2 antibodies in comparison with neutralization activity. EBioMedicine. 2020;62:103101. doi:10.1016/j.ebiom.2020.103101
2. Bonfante F, Costenaro P, Cantarutti A, et al. Mild SARS-CoV-2 Infections and Neutralizing Antibody Titers. Pediatrics. 2021;148(3):e2021052173. doi:10.1542/peds.2021-052173

© 2022 Di Chiara C et al. *JAMA Network Open.*

eFigure 1. Family Clusters of COVID-19 Observed From April, 1st to August 31st, 2021, at the COVID-19 Follow-up Clinic of Our Institution

Blue: whole cohort of enrolled subjects; green: individuals excluded from the analysis; orange: confirmed COVID-19 cases.

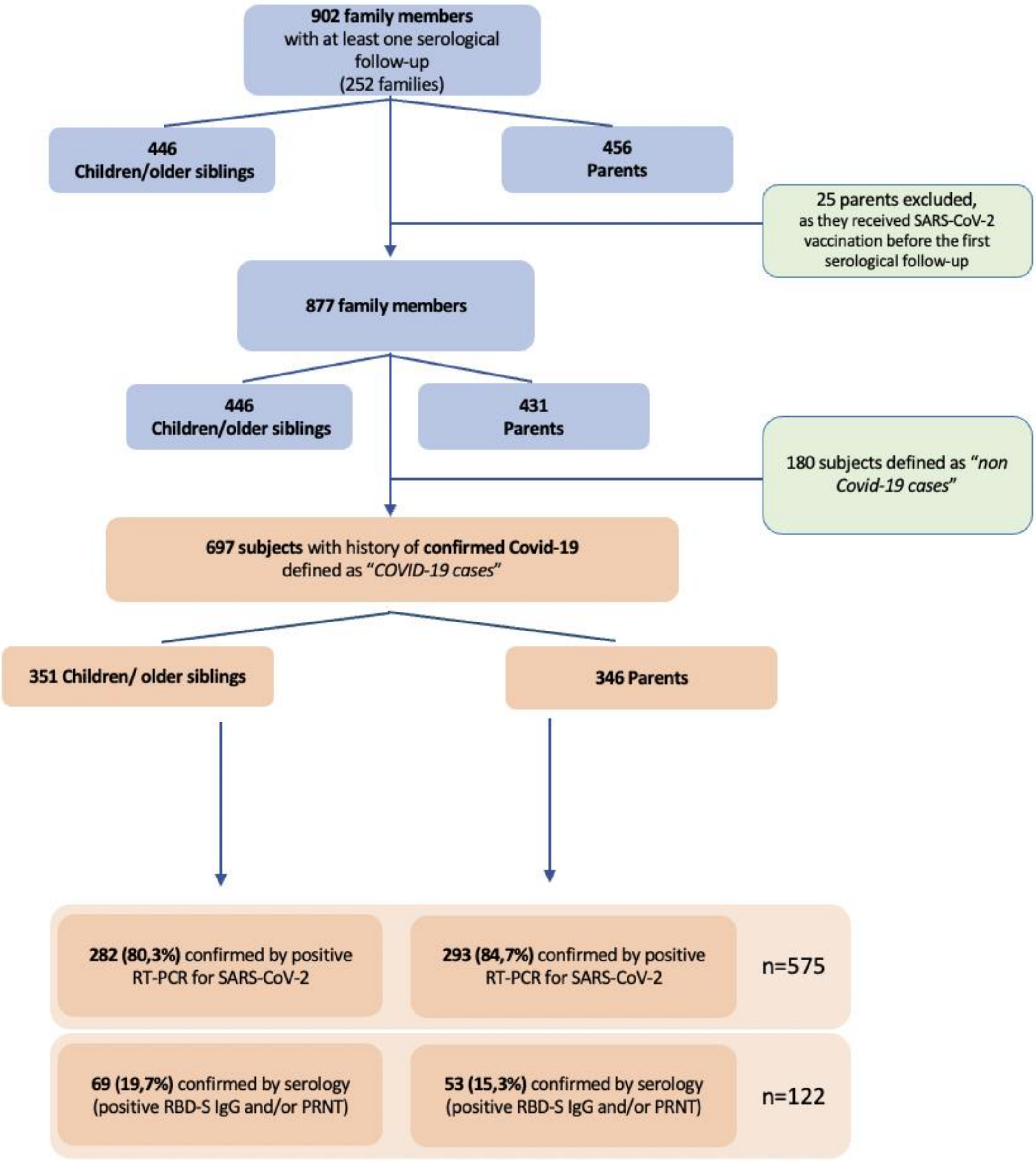

eFigure 2. Criteria for the Definition of the Baseline Time for COVID-19 Cases

© 2022 Di Chiara C et al. *JAMA Network Open.*

For symptomatic cases the baseline of infection was defined as the onset of symptoms or the date of first positive SARS-CoV-2 molecular assay; while for asymptomatic cases as the date of the first positive molecular assay or by the family outbreak temporal sequence.

Only for 67 (9,6%) asymptomatic cases with negative or not performed NPs, but with evidence of SARS-CoV-2 seropositivity, the *baseline time* was identified as the symptoms' onset of the first symptomatic family member.

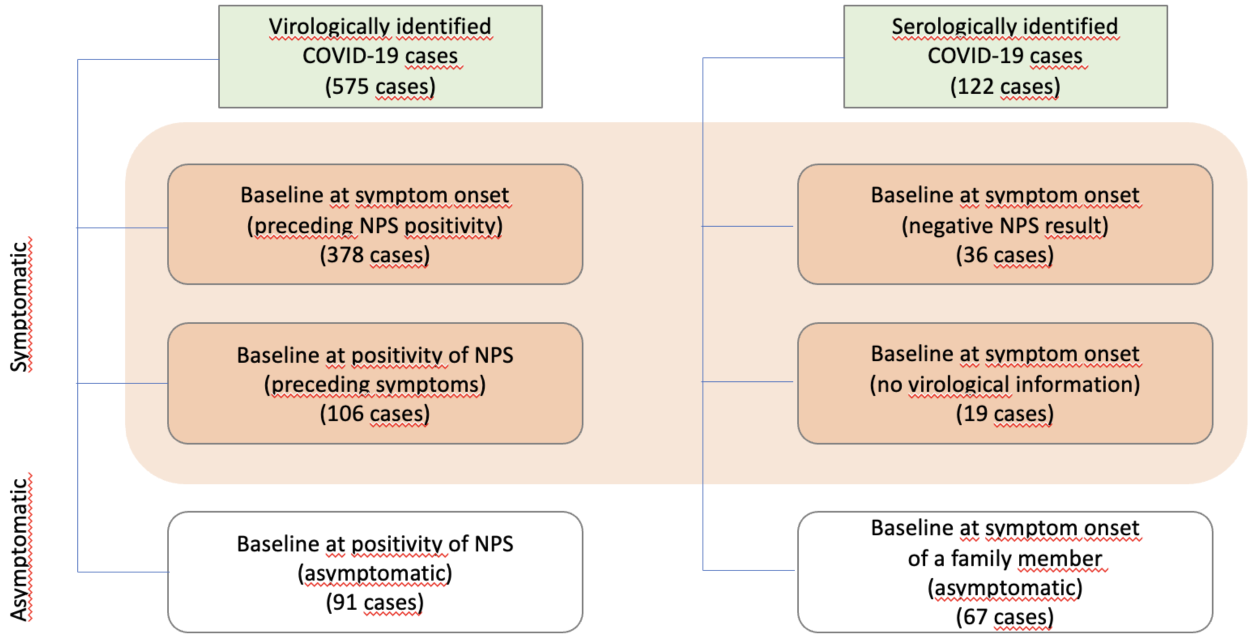

eFigure 3. Distribution of S-RBD IgG Samples According to Time of Collection and Age Classes (n=769)

Younger patients presented higher levels of Abs across all time points of samples collection.

S-RBD IgG levels are reported in log2 scales. The dotted lines at 4.33 kBAU/L correspond to the assay cut-off for discriminating positive from negative samples.

© 2022 Di Chiara C et al. *JAMA Network Open.*

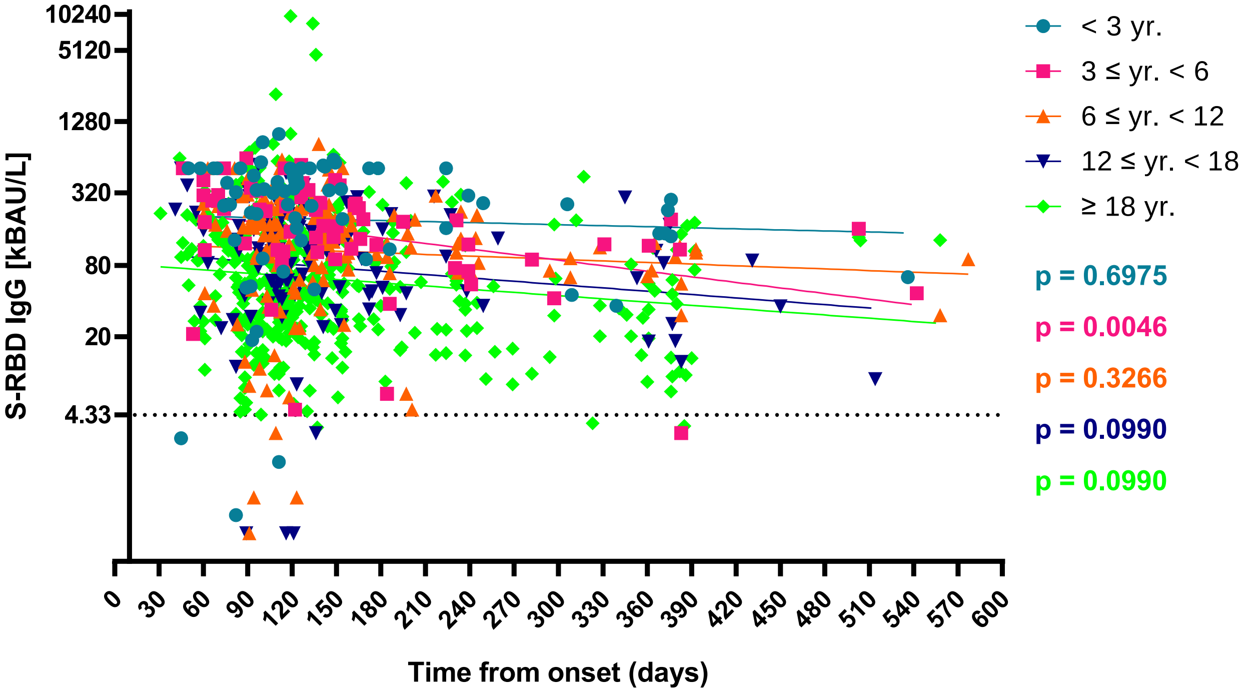

eFigure 4. Distribution of S-RBD IgG Samples According to Age Classes

Younger children presented a significantly higher levels of Abs than adults.

S-RBD IgG levels are reported in log2 scales. The dotted lines at 4.33 kBAU/L correspond to the assay cut-off for discriminating positive from negative samples.

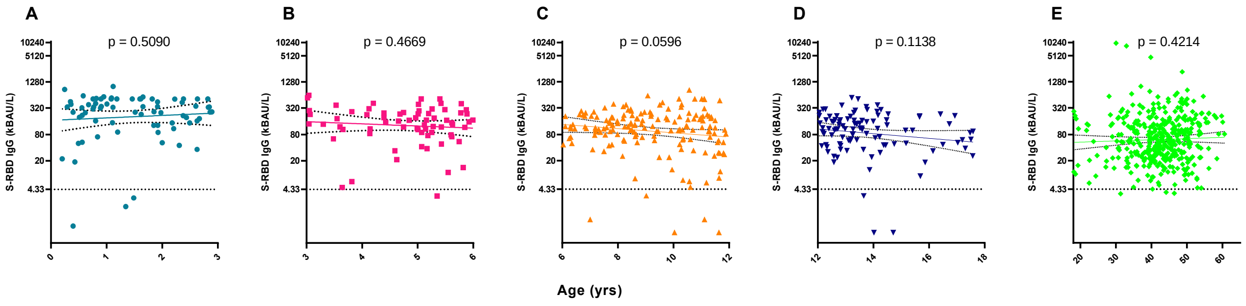

© 2022 Di Chiara C et al. *JAMA Network Open.*

eTable. Demographic and Clinical Characteristics of the Analyzed Population, Overall (n=876) and Stratified by Familiar Status as Children or Older Siblings (n=446) and Parents (n=431)

	**OVERALL**					**CHILDREN/OLDER SIBLINGS**					**PARENTS**				
	**COVID-19 negative**		**COVID-19 positive**		*p-value* §	**COVID-19 negative**		**COVID-19 positive**		*p-value* §	**COVID-19 negative**		**COVID-19 positive**		*p-value* §
	(n=179)		(n=697)			(n=94)		(n=351)			(n=85)		(n=346)		
**Female** (n, %)	83	(46.4)	321	(46)	0.94	41	(43.6)	155	(44.2)	0.93	42	(49.4)	166	(48)	0.81
**Age** (mean, SD)	27.1	±18.6	25.4	±18.0	0.28	10.4	±5.9	8.6	±5.1	0.005	45.6	±6.1	42.5	±7.1	0.0003
**Age classes** (n, %):															
< 3 years	4	(2.2)	55	(8)	0.04	4	(4.3)	55	(15.7)	0.0002	-	-	-	-	-
3 ≤ years < 6	19	(10.6)	47	(6.7)		19	(20.2)	47	(13.4)		-	-	-	-	
6 ≤ years < 12	37	(20.7)	141	(20.2)		37	(39.4)	141	(40.2)		-	-	-	-	
12 ≤ years < 18	21	(11.7)	94	(13.5)		21	(22.3)	94	(26.8)		-	-	-	-	
≥ 18 years	98	(54.8)	360	(51.6)		13	(13.8)	14	(4)		85	(100)	346	(100)	
**Symptomatic** (n, %):	4	(2.2)	540	(77.5)	<0.0001	2	(2.1)	241	(68.7)	<0.0001	2	(2.3)	299	(86.4)	<0.0001
**WHO classification***															
(n, %):															
Asymptomatic	-	-	157	(22.5)	-	-	-	111	(31.9)	-	-	-	47	(13.6)	-
Mild	-	-	516	(73.9)		-	-	231	(65.3)		-	-	285	(82.4)	
Moderate / severe	-	-	14	(2)		-	-	1	(0.3)		-	-	13	(3.8)	
Critical	-	-	1	(0.1)		-	-	0	(0)		-	-	1	(0.3)	
MIS-C	-	-	9	(1.3)		-	-	9	(2.5)		-	-	0	(0)	
**comorbidities:**															
No						82	(87.2)	290	(82.6)	0.28	72	(84.7)	286	(82.4)	
Yes**						12	(12.8)	59	(17.4)		13 (15.3)		61	(17.6)	

§ T student test, χ2 test, Fisher exact test where appropriate. *WHO, World Health Organization; MIS-C, Multisystem Inflammatory Syndrome in Children. **The following co-morbidities were found among 59 COVID-19 positive children: premature birth (n=6), asthma (n=15), allergy (n=6), congenital heart disease (n=6), rheumatological disease (n=3), neuro-epileptic disease (n=5), metabolic disease (n=1), kidney/ureteral disease (n=4), endocrinological disease (n=2), gastrointestinal disease (n=4).

© 2022 Di Chiara C et al. *JAMA Network Open.*
